# Mechanistic Insights into the Uptake and Adsorption Kinetics of Kiperin Dihydrolyzed Collagen in Radish Seedlings

**DOI:** 10.3390/life16101709

**Published:** 2026-10-12

**Authors:** Serap Saglam, Fatih Bildik, Lutfiye Karcioglu Batur, Ahsen Pektas, Buse Aslan, Ismail Kocacaliskan

**Affiliations:** 1 Department of Biology, Division of Botany, Faculty of Science, Istanbul University, 34134 Istanbul, Türkiye; sercag@istanbul.edu.tr; 2 Department of Food Engineering, Faculty of Chemical and Metallurgical Engineering, Istanbul Technical University, Maslak, 34469 Istanbul, Türkiye; 3 Department of Molecular Biology and Genetics, Faculty of Engineering and Natural Sciences, Biruni University, 34015 Istanbul, Türkiye; 4 Biruni University Research Center (B@MER), Biruni University, 34015 Istanbul, Türkiye; 5 Kiperin Pharmaceutical and Food Industry and Trade Ltd., 34307 Istanbul, Türkiye; ahsennzerin@gmail.com (A.P.); busseaaslan@gmail.com (B.A.)

**Keywords:** radish, hydrolyzed collagen, amino acid uptake, adsorption kinetics, adsorption isotherms

## Abstract

This study investigated the root uptake and adsorption kinetics of Kiperin dihydrolyzed collagen (KDHC), an organic nitrogen source rich in amino acids and low-molecular-weight peptides, in radish (*Raphanus sativus* L.) seedlings grown in perlite. KDHC was applied at 0.1, 1.0, and 10 g L^−1^ to 7-day-old seedlings. Directly measured solution depletion and tissue accumulation were distinguished from processes inferred from model fits, such as apoplastic adsorption and transport between organs. Amino acid depletion from the root medium increased with the applied concentration, and the Freundlich, Langmuir, Dubinin–Radushkevich, and Flory–Huggins isotherm models described amino acid binding to the root surface. Depletion from the root medium followed pseudo-first-order kinetics (R^2^ = 0.94–0.99), reflecting rapid apoplastic surface adsorption within 60 min. In an ex vivo leaf disk assay, cellular accumulation in leaf tissue followed the pseudo-second-order model (R^2^ = 0.9881), suggesting a carrier-mediated mechanism. Differences among amino acids were interpreted in relation to their charge states at pH 5.8 and their molecular size. Root-to-shoot translocation was not traced directly and should be confirmed using isotope-labelled amino acids. These findings indicate that KDHC-derived amino acids are readily available to radish roots and may serve as an organic nitrogen source.

## 1. Introduction

Nitrogen is the most important nutrient that contributes to plant growth and development [1]. Due to its presence in the structure of proteins, amino acids, nucleic acids, chlorophyll, and numerous metabolites, it is of critical importance for the sustainability of plant production [2,3,4]. In traditional agricultural systems, the largest share of plants’ nitrogen requirement is met by inorganic fertilizers in the form of nitrate and ammonium. In addition, it is known that plants can directly utilize not only inorganic nitrogen but also organic nitrogen compounds. Amino acids, peptides, and proteins constitute the majority of the organic matter structure of soil [5]. For many years, it was thought that these compounds must first be mineralized by microbes into ammonium or nitrate before they could be used by plants. Recent studies have shown that many plant species are able to take up amino acids directly via their roots in their unaltered form [3,6,7]. Plants do not merely use amino acids as a source of nitrogen; they also utilise them to promote growth, regulate metabolism and enhance tolerance to stress conditions [8,9,10]. Amino acids are easily taken up by the roots via specialised carrier proteins located in the plasma membrane. More than 100 potential amino acid transporters have been identified in Arabidopsis thaliana [3]. Most of these belong to families such as amino acid permeases (AAP) and lysine-histidine transporters (LHT) [11,12]. Amino acids disappearing from the external solution via root–surface interactions subsequently pass through the cortex and endodermis, potentially entering the vascular system for translocation, while maintaining a clear methodological distinction between measured solution depletion and inferred internal transport [10,13,14]. The amino acids detected in shoot tissues are distributed within the plant via long-distance transport systems. Amino acids taken up by the roots are transferred to the xylem during transpiration, delivered to the shoots in this way, and subsequently redistributed to developing tissues via the phloem [12,14,15].

The accumulation of amino acids in shoot tissues is attributed to their rapid systemic translocation via the vascular system without undergoing extensive root storage or immediate local metabolic conversion. This is consistent with studies indicating that amino acids move easily and may also act as signalling molecules [3,4]. However, in studies on the long-distance transport and redistribution of amino acids in plants, the mechanism has not yet been fully explained. In recent years, interest in amino acid and peptide-based biostimulants has increased significantly. These products support plant growth by promoting root formation and enhancing the efficiency of nutrient uptake [8,16]. In this context, the dihydrolyzed collagen used in our study stands out as an important organic nitrogen source, containing high levels of amino acids and low molecular weight peptides. Hydrolyzed collagen is a product formed by the enzymatic or chemical breakdown of collagen proteins, generally consisting of peptides with a molecular weight of 3–6 kDa [17,18]. Due to the amino acids and biologically active peptides it contains, it is considered a potential biostimulant in plant nutrition applications. The characteristics of plant growth media play a very important role in investigating plants’ ability to use organic nitrogen sources. Perlite is a substrate widely used in controlled cultivation systems thanks to its high porosity, low cation exchange and adsorption capacity, and chemically inert structure [19,20]. Perlite’s low adsorption capacity allows amino acids and peptides to reach the root without being retained by the medium, providing an advantage to the plant. Amino acids taken up by the roots are then transported upward according to enzymatic and physiological principles. Radishes have a rapidly developing root system. Due to its high nutrient uptake capacity and short growth period, it is a commonly used model plant in studies of root physiology and nutrient uptake [21,22]. Its robust root system makes it an extremely suitable research material for examining the uptake of low-molecular-weight organic compounds. Although it is now well known that amino acids can be taken up directly by roots, information regarding the uptake of amino acids and peptides derived from hydrolyzed collagen by plants, their transport to the leaves, and the relationship between these processes and physicochemical properties is still limited. In particular, the mechanism underlying the kinetic behaviors of amino acid uptake, depending on the applied concentration, and their translocation from roots to leaves has not been sufficiently elucidated. In our literature review, we did not encounter any study on the uptake of hydrolyzed collagen from soil to roots and its translocation from roots to leaves in radish plants. Therefore, this study aimed to characterize the root uptake kinetics and adsorption behavior of amino acids derived from KDHC (Kiperin dihydrolyzed collagen) applied at different concentrations to radish seedlings grown in perlite, and to assess the capacity of leaf tissue to take up these amino acids. In this way, we aimed to obtain new insights into the mechanisms of organic nitrogen utilization by plants.

## 2. Materials and Methods

### 2.1. Experimental Design Image

**Figure 1:**
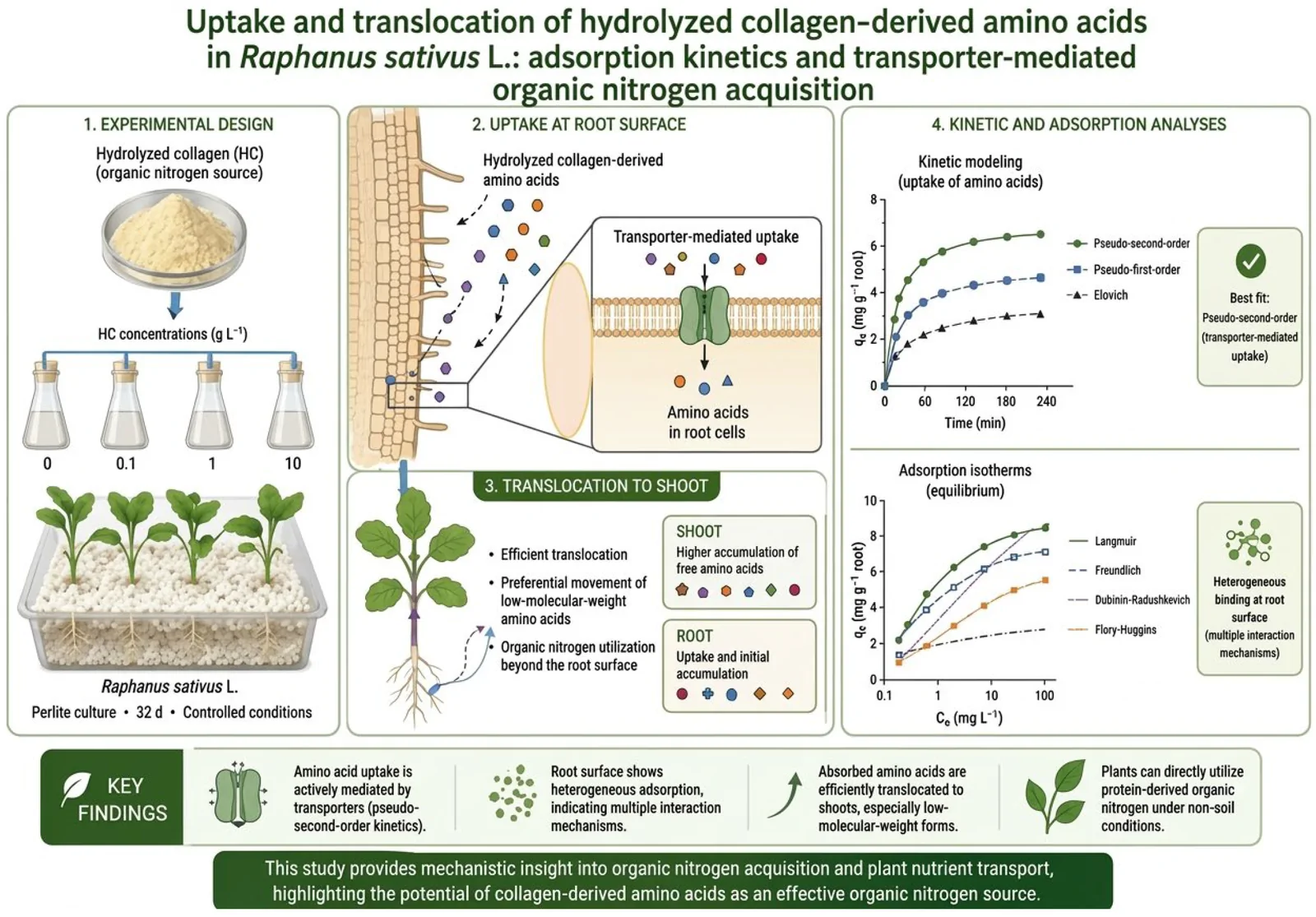
Experimental design and conceptual workflow of the study. Amino acids derived from KDHC were applied to radish seedlings grown in perlite under controlled conditions. (1) summary of the experimental setup and dose applications, (2) root surface uptake mechanism, (3) root-to-shoot translocation, and (4) kinetic and isotherm models explaining uptake behavior. The experiment followed a Completely Randomized Design (CRD) with 15 plants per treatment (*N* = 15, distributed across 5 independent pots with 3 plants per pot). Determinations were conducted using 3 biological replicates and 3 technical replicates (*n* = 9), and the entire experiment was independently repeated twice.

The experimental setup was carefully designed to evaluate the root uptake and translocation dynamics of Kiperin dihydrolyzed collagen in radish seedlings grown under controlled environmental conditions. The overall workflow and dose applications are systematically illustrated below in Figure 1.

### 2.2. Plant Material and Experimental Conditions

In our study, radish seeds were obtained from Intfa Tarım (Catalog no: IF-SSTP006, Intfa, Konya, Türkiye). Seeds were germinated under controlled conditions; those with similar root lengths were selected for growth and transferred to plastic trays (7 cm × 7 cm × 9 cm) filled with agricultural perlite of medium particle size as the cultivation substrate. The seedlings were transferred to a controlled climate chamber set to 25 ± 2 °C, relative humidity 60–70%, and photoperiod of 16 h light/8 h dark (16L:8D). Prior to treatments, seedlings were given a 7-day adaptation period in the perlite medium.

### 2.3. Dihydrolyzed Collagen Applications

Dihydrolyzed collagen (obtained from Kiperin firm, Istanbul, Türkiye) was used as the organic nitrogen source in the study. The application solution of dihydrolysed collagen was prepared by dissolving it in distilled water at concentrations of 0.1 g L^−1^, 1.0 g L^−1^ and 10 g L^−1^. The control group received distilled water (D.W.). Applications were given in equal volumes to the root zone every other day. The volume (ml) applied increased according to plant water needs as root growth progressed. Plant growth was monitored regularly throughout the experiment.

### 2.4. Harvest and Sampling

To establish a clear analytical framework, three distinct processes were evaluated: (1) Solution depletion refers to the directly measured decrease in amino acid concentration in the incubation medium over time; (2) Root apoplastic adsorption represents surface binding evaluated via isotherm models (Langmuir, Freundlich, D–R); and (3) Tissue accumulation designates the amino acid content measured inside tissue extracts following desorption washing with 0.25 mM CaCl_2._ Processes (1) and (3) were measured directly, whereas adsorption mechanisms and transport between organs were inferred from model fits and the literature.

Plants were harvested 32 days after the start of applications. During harvest, seedlings were carefully removed from the perlite medium, and perlite residue on roots was washed away with distilled water. Root and leaf tissues were separated and sampled for evaluations. The roots were carefully dried using soft paper, then immersed in a 0.25 mM CaCl_2_ solution to separate the adsorbed compounds, dried again, and then immediately placed in an amino acid incubation solution consisting of a standardized 16-amino acid blend representing the profile of Dihydrolyzed Collagen Peptides (KDHC, Type I and Type III) dissolved in ultrapure water. Each root system was placed in 5 mL of incubation solution in a 10 mL tube. Each tube containing roots was weighed quickly at the start and finish of the experiment to monitor evaporation losses. During the adsorption experiments, the test solution was aerated using a vacuum pump coupled with a sterile filter (0.22 μm) fitted to a 1.0 mm diameter needle. To ensure solution homogeneity while preventing mechanical damage to root hairs and epidermal tissues, low-speed magnetic stirring (50 rpm) with a floating stir bar setup was employed. Every adsorption experiment lasted one hour. Solution depletion was recorded as the decrease in amino acid concentration in the incubation solution. After placing the plant roots in the solution, an initial sample (100 μL) was collected from the incubated solution, and this sampling was replicated every 20 min. The samples were stored on ice and kept in a −20 °C freezer for analysis. Following the uptake measurement, the roots were washed again with 0.25 mM CaCl_2_. The maximum root length, shoot length, and the number of leaves were noted. The shoots and roots were then divided, left on ice for 1 h, and preserved in the freezer. The shoots and roots were later dried at 50 °C for 24 h and then weighed.

### 2.5. Leaf Tissue Preparation and Cellular Uptake Kinetics

To examine the cellular uptake and membrane transport dynamics of collagen-derived amino acids in aerial parts, uniform leaf disks (5 mm in diameter) were punched from freshly harvested leaves using a precision cork borer. Following excision, the disks were rinsed three times with ultrapure water to eliminate cellular debris originating from the cut edges. The prepared tissue samples were then incubated in buffered amino acid test solutions (control/KDHC-treated 0.1, 1.0, and 10 g L^−1^, buffer, pH:5.8) at 25 °C under continuous illumination. To accurately differentiate true intracellular absorption and apoplastic binding from superficial residue, leaf samples were collected at designated time intervals (0–120 min) and subjected to two consecutive 5 min desorption washes using ice-cold 0.25 mM CaCl_2_ prior to subsequent homogenization and extraction. This ex vivo assay reflects the uptake capacity of leaf tissue from an external solution; it does not directly measure amino acids translocated from the roots.

### 2.6. Analytical Methodology and Method Validation

Total free amino acid concentrations in solution and tissue extracts were determined colorimetrically using a modified Ninhydrin assay at λ = 570 nm against L-leucine calibration standards (R^2^ > 0.999). Individual amino acid profiles in hydrolyzed collagen and plant tissues were resolved and quantified using Reversed-Phase High-Performance Liquid Chromatography (RP-HPLC; Agilent 1260 Infinity system) equipped with a C18 column (4.6 mm × 150 mm, 3.5 µm) and fluorescence detection. Pre-column derivatization was carried out using o-phthalaldehyde (OPA) for primary amino acids and 9-fluorenylmethyl chloroformate (FMOC) for secondary amino acids. L-norvaline was explicitly incorporated as an internal standard across all chromatographic runs to ensure high analytical precision and recovery rates. Limits of Detection (LOD) and Quantification (LOQ) ranged from 0.01 to 0.05 µmol L^−1^ and 0.03 to 0.15 µmol L^−1^, respectively.

### 2.7. Determination of Amino Acid Uptake

The diverse amino acids found in hydrolyzed collagen peptides served as a guide in selecting the types of amino acids and their concentration ranges to be used in amino acid uptake studies. Our aim is also to include amino acids known to have a relatively high conversion rate in soil, and that exhibit different characteristics in terms of the charge on their side chains at the working pH. The concentrations of individual amino acids in the solution to which perlite was added range from 0.1, 1.0, and 10 g L^−1^. The incubation solutions used in the amino acid uptake study were prepared as a mixture consisting of equal concentrations of a 16-amino acid blend containing KDHC (Hydrolyzed Collagen Peptides Type I and Type III) dissolved in ultrapure water. All incubation solutions contained a 0.5 mM concentration of CaCl_2_ to preserve the membrane stability of the root cells.

At the start of the experiments, the pH of the solution ranged between 5.0 and 6.0. The ability to uptake amino acids was tested in pure amino acid incubation solutions at three concentrations of each amino acid: 0.1, 1.0, and 10 g L^−1^. The concentrations measured at the beginning of each experiment were used for calculations. Determination of amino acids transferred to plants from perlite medium.

Amino acid content was analyzed in root extracts of plants after flow measurement in 0.25 mM CaCl_2_. Frozen roots were ground to a fine powder in a ball mill. Ground samples (10–20 mg) were extracted in 1 mL 0.05 M HCl under shaking at 4 °C for 1 h. The supernatant (200 μL) was stabilized with 50 μL 5 μM homocysteine acid after centrifugation at 5000 rpm for 10 min, and amino acids were determined as described in Section 2.6.

### 2.8. Kinetics of the Amino Acids Sorption

To assess the activity of the amino acids, batch kinetic experiments were carried out using highly diluted solutions [0.05 µg amino acid/100 µL PBS-water (10 per cent by volume) mixture]. For this purpose, roots (0.01 g) were soaked in distilled water (0.05 mL) and added to the amino acid solution (100 µL). The mixtures were gently stirred at low speed (50 rpm) with a floating stir bar, as described in Section 2.4, and samples (50 µL) were taken at 0, 20, 40, and 60 min for analysis as described in Section 2.6. At the end of each incubation, root integrity was checked visually under a stereomicroscope, and no damage to root hairs or epidermal tissues was observed.

### 2.9. Kinetic Rate Equations

The pseudo-first-order kinetic model given by Lagergren as follows:

ln (q_e_ − q_t_) = ln q_e_ − k_1_ t (1)

The linear form pseudo-second-order kinetic model is based on chemical adsorption and can be expressed as follows:

t/q_t_ = 1/k_2_ q_e_ + 1/q_e_ t (2)

Intra particular rate equation;

q_t_ = k_i_ t_1/2_ + c (3)

where:

*q_e_*: Adsorption capacity at equilibrium (mg g^−1^), representing the amount of amino acid adsorbed per unit mass of plant tissue at equilibrium.

*q_t_*: Adsorption capacity at time t (mg g^−1^), representing the amount adsorbed at any specific time point.

*k*_1_: Pseudo-first-order rate constant (h^−1^).

*k*_2_: Pseudo-second-order rate constant (g mg^−1^ h^−1^).

t*t*: Incubation/adsorption time (h).

*k_i_*: Intra-particle diffusion rate constant (mg g^−1^ h^−1/2^).

*c*: Intercept related to the thickness of the boundary layer (mg g^−1^).

### 2.10. Adsorption Isotherms Models

Six models were applied for adsorption isotherm studies, respectively Freundlich, Langmuir, BET, Temkin, Dubinin–Radushkevich (D-R) and Flory–Huggins (F-H) [23,24]. Freundlich model suggests that first the adsorbate amount in the adsorbent increases with increasing concentration without predicting saturation. In Table 1, qe, the amount of matter adsorbed by the adsorbent at equilibrium; ce, the concentration of adsorbed matter at equilibrium; KF, adsorption capacity: n, adsorption intensity. Langmuir model explains that a monomolecular layer has formed and qe has shown a boundary value, because the surface of the adsorbent is covered with the adsorption of gas or liquid molecules. In Table 1, qmax, maximum adsorption capacity of the adsorbent at monolayer; KL, Langmuir constant. In BET model, before the surface of the adsorbent is covered by a monomolecular layer, a multimolecular layer forms on this surface. In Table 1, k, a constant at any temperature; Cs, concentration of the saturated solution for adsorbate. Dubinin–Radushkevich (D-R) model suggests that the adsorbed monolayer shows a multilayer character to distinguish physical from chemical adsorption. The Polanyi constant (ε) is presented in Table 1. This constant is calculated as RT ln (1 + 1/ce). KDR is D-R isotherm constant. According to the equation in Table 1, the adsorption energy (E) is found from this isotherm constant. The Flory–Huggins (F-H) model explains the degree of surface coverage. This degree is found from the equation (Q = 1 − Ce/Co). Co is the initial concentration. KFH is a constant. This constant is used to calculate the Gibbs free energy change (ΔG^0^) as seen in Table 1. Linearized Freundlich;

ln q_e_ = ln KF + n ln c_e_ (4)

where KF is the Freundlich adsorption capacity constant (mg g^−1^ (L mg^−1^)^n^) and n is the Freundlich exponent (adsorption intensity), with 0 < n < 1 indicating favorable adsorption.

Langmuir;

c_e_/q_e_ = (1/q_max_KL) + (c_e_/q_max_) (5)

where:

qmax: Maximum monolayer adsorption capacity (mg g^−1^).

KL: Langmuir equilibrium constant (L mg^−1^)

BET;

c_e_/q_e_(c_s_ − c_e_) = 1/(q_m_·k) + [(k − 1)/(q_m_·k)](c_e_/c_s_) (6)

where cs is the saturation concentration of the adsorbate (mg L^−1^), qm is the monolayer adsorption capacity (mg g^−1^), and k is the BET constant related to the energy of surface interaction.

Temkin;

q_e_ = Bln α + Bln c_e_ (7)

where; B = RT/bT, where R is the universal gas constant (8.314 J mol^−1^ K^−1^), T is the absolute temperature (K), and bT is the Temkin constant related to the heat of adsorption (J mol^−1^)

In this isotherm, interactions among the adsorbed substances are taken into account. The Temkin isotherm constant, α, is the equilibrium binding constant (L g^−1^). The values of B and α are calculated from the slope and intercept, respectively, of the linear plot of qe versus ln ce.

The Dubinin–Radushkevich Isotherm (D-R);

This isotherm is generally expressed as follows:

ln x_e_ = ln x_m_ − K_DR_ ε^2^ (8)

ε = RT ln (1 + 1/c_e_) (9)

where ce is adsorbate concentration in solution at equilibrium (mol L^−1^).

Radushkevich and Dubinin have reported that the characteristic sorption curve is related to the porous structure of the sorbent Xm [25]. The constant, KDR, is related to the mean free energy of adsorption per mole of the sorbate as it is transferred to the surface of the solid from infinite distance in the solution, and this energy can be computed using the following relationship:

(10) $$E\;=\;1/\sqrt{2\mathrm{KDR}}$$

When ln xe is plotted against ε^2^, a straight line with slope −KDR and intercept ln xm is obtained.

where:

xe: Amount of adsorbate in the solid phase at equilibrium (mol g^−1^)

xm: Theoretical saturation capacity (mol g^−1^)

KDR: D-R isotherm constant (mol^2^kJ^−2^)

ε: Polanyi potential

E: Mean free energy of adsorption (kJ mol^−1^)

The Flory–Huggins Isotherm (F-H);

The F-H model was chosen in order to account for the degree of surface coverage characteristics of the adsorbate on the adsorbent. The linear form of the F-H equation is represented by,

log Q/c = log K_FH_ + n log (1 − Q) (11)

where c_0_ and ce are described before, Q = (1 − ce/c_0_) is the degree of surface coverage, n is the number of adsorbates occupying adsorption sites, KFH is the equilibrium constant. When log (Q/c) is plotted against log (1 − Q), a straight line with slope n and intercept logKFH is obtained. Furthermore, the equilibrium constant, KFH, was used to compute the Gibbs free energy of spontaneity (ΔG^0^). The Gibbs free energy of spontaneity is related to the equilibrium constant as follows

ΔG^0^ = −2.303 RT log K_FH_ (12)

where R is the universal gas constant (8.314 J mol^−1^ K^−1^) and T is the absolute temperature (K).

### 2.11. Data Processing and Statistics

The uptake rate of each amino acid was calculated from the linear regression between the amino acid concentration in solution and experimental time (0–120 min). Each regression was based on five sampling times. Uptake rates and the Langmuir and Freundlich isotherms were analyzed linearly; concentration dependence followed asymptotic second-order reaction kinetics. The relationship between uptake rate and initial concentrations was described by polynomial regression. For control experiments, linear regression was used.

## 3. Results

### 3.1. Uptake of Dihydrolyzed Collagen Peptides by Roots and Assessment of the Transport Mechanism

As dihydrolyzed collagen dissolves in the perlite medium, low-molecular-weight amino acids and peptides emerge. The availability of these for uptake by roots was evaluated based on currently defined organic nitrogen uptake mechanisms. It was determined that amino acids in the dihydrolyzed collagen peptides applied to perlite were efficiently taken up by radish roots. Amino acid uptake increased in parallel with the application concentration. Examining the relationship between the amounts of amino acids bound to the root surface and equilibrium concentrations revealed that the adsorption behavior tended toward saturation. It was determined that the Freundlich, Langmuir, Dubinin–Radushkevich (D-R), and Flory–Huggins (F-H) isotherm models provided suitable results in describing amino acid adsorption to the root surface. The obtained adsorption isotherms showed that amino acids bind to the root surface with high affinity.

### 3.2. Adsorption Studies

**Figure 2:**
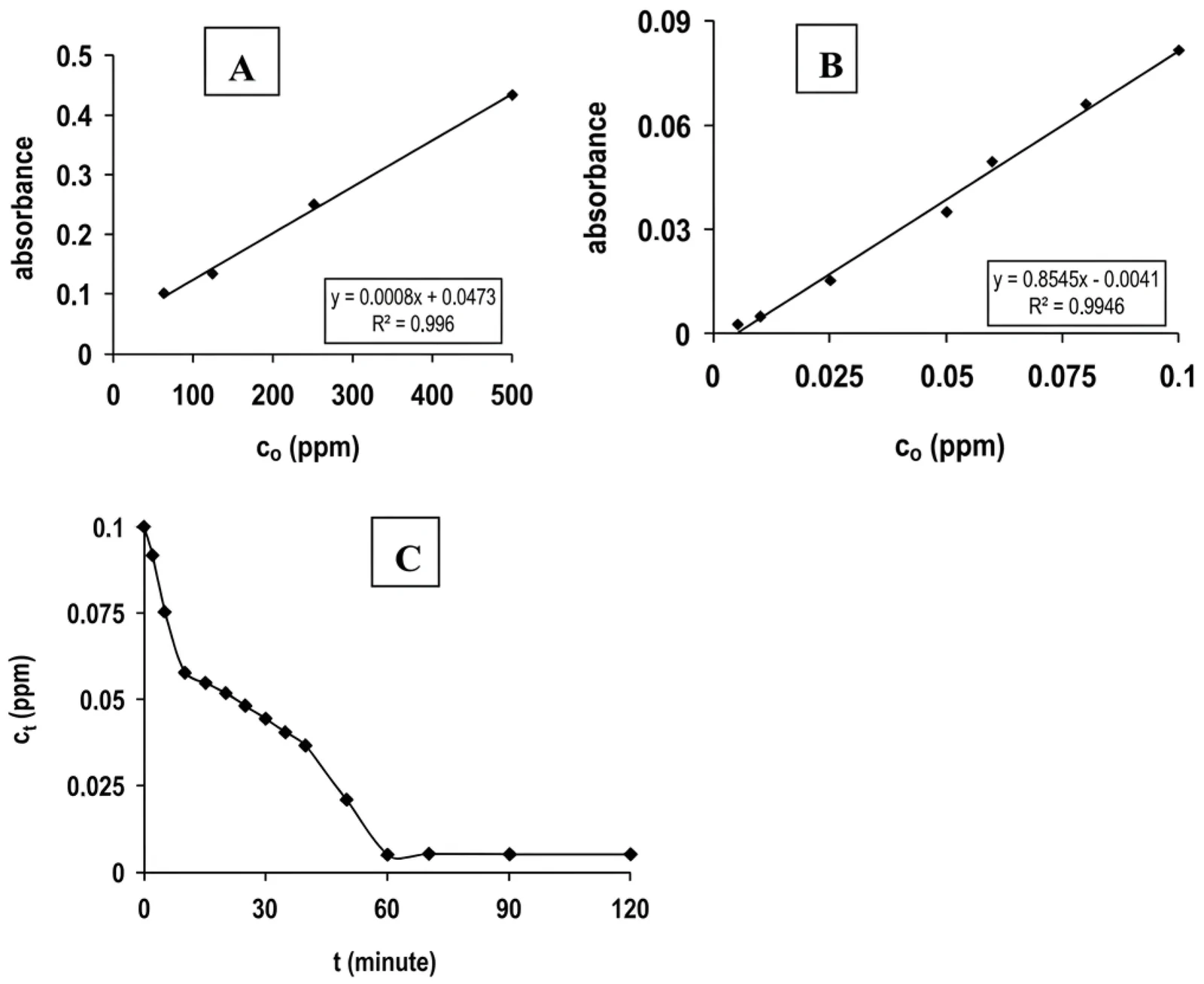
Calibration curves of amino acids at higher (**A**) and lower (**B**) concentrations; time-dependent concentration depletion of amino acids (1 g L^−1^) on roots at 25 °C (**C**).

As a result of graphing qe values against equilibrium concentration (ce), it was determined that the adsorption exhibits a type L isotherm behavior (Figure 2). This indicates that amino acids have a strong binding tendency to the root surface.

The n value obtained in Freundlich isotherm analysis, being between 0 and 1, indicates that the adsorption process is favorable. The maximum adsorption capacity (qmax) calculated from the Langmuir isotherm model was determined as 91.2 mg g^−1^. The maximum adsorption capacity calculated from the BET isotherm was found to be 90.0 mg g^−1^. The correlation coefficients (R^2^) associated with Freundlich, Langmuir, and BET models ranged from 0.75–0.98, indicating that the experimental data were well-explained by these models. The maximum adsorption capacity calculated from the Dubinin–Radushkevich isotherm was determined as 41.1 mg g^−1^. The adsorption energy (E) value obtained from this model was calculated as 0.091 kJ mol^−1^ (for root–surface interaction). Gibbs free energy change (ΔG) was calculated from Flory–Huggins isotherm analysis, and the thermodynamic properties of the adsorption process were determined. The data obtained indicated that the adsorption process occurs spontaneously.

### 3.3. Adsorption Kinetics

**Figure 3:**
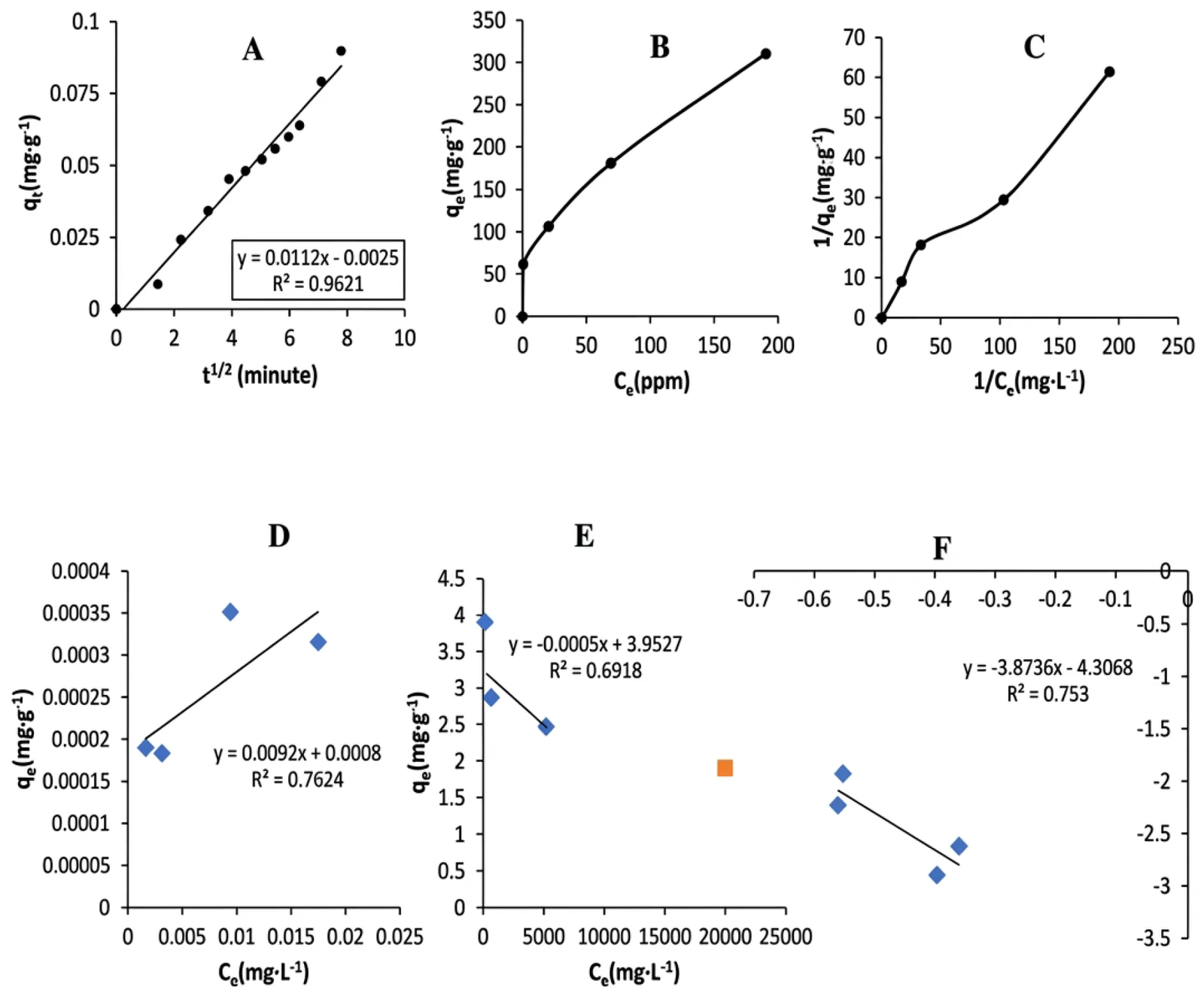
Kinetics equations of amino acids at 25 °C on roots. Pseudo-first-order, pseudo-second-order model, intra-particle diffusion model (**A**); Freundlich, Adsorption isotherm curve of amino acids on root (**B**); Langmuir Adsorption isotherm curve of amino acids on root (**C**); BET Adsorption isotherm curve of amino acids on root (**D**); D-R Adsorption isotherm curve of amino acids on root (**E**); Temkin Adsorption isotherm curve of amino acids on root (**F**).

The adsorption kinetics of dihydrolyzed collagen peptide solutions, prepared in the 0.1–10 g L^−1^ concentration range, on radish roots were investigated. Results from the time-dependent concentration changes showed that adsorption equilibrium was reached within approximately 60 min. For spectrophotometric analyses, standard solutions in the 62.5–500 ppm concentration range were used, and calibration curves were generated. The coefficients of determination (R^2^) for the linear calibration curves and kinetic regressions ranged between 0.85 and 0.99 across all evaluated concentration ranges. Kinetic studies of the adsorption of hydrolyzed collagen peptide solution with a concentration of 0.1–10 g L^−1^ on radish roots in perlite were performed at 25 °C, and the concentrations (ct) were calculated from the absorbance values measured at specified time intervals using standard curves. From the plot of concentration versus time, as shown in Figure 3, the time to reach equilibrium was accepted as 60 min.

In this study, the pseudo-first-order rate equation, pseudo-second-order rate equation, and intra-particle diffusion model were used in calculations (Figure 3). Parameters for these equations are shown in Table 1.

Here, the experimental equilibrium adsorbed amount qe was found to be 0.0899 mg g^−1^. The pseudo-first-order rate equation had the best fit to this value and the highest regression value. The plot of qt versus t_1/2_ showed that the intra-particle rate occurred in a single step, with the regression value (R^2^) of 0.98 as indicated in Table 1. Accordingly, adsorption from the aqueous phase onto the root surface is considered to occur rapidly as surface adsorption within 60 min. The high correlation coefficient obtained (R^2^ ≈ 0.98) revealed that amino acids are rapidly adsorbed onto the root surface.

### 3.4. Adsorption Isotherms

In isotherm studies conducted at 25 °C, it was determined that the adsorption behavior of amino acids on the root surface within the concentration range of 62.5–500 ppm is consistent with the L-type isotherm according to the Giles classification. This result demonstrates that amino acids exhibit a high binding affinity to the root surface. Accordingly, this graph represents a situation where concentrations are very high, and the adsorbate (amino acids) shows a very strong tendency to bind to the adsorbent (root). At amino acid concentrations ≤ 0.099 ppm, the solution is very dilute, and since the dissolved substance is completely adsorbed, no measurable amount remains. In the Freundlich isotherm, *n* < 1 indicates that the adsorption is favorable [26]. The KF adsorption capacity constant for amino acids was calculated as 1.22 mg g^−1^ (L mg^−1^)^n^, as shown in Table 1. According to the Langmuir isotherm, the maximum amount of substance adsorbed in a monolayer, qmax, was determined to be 91.2 mg g^−1^. The large values of these parameters indicate that hydrolyzed collagen peptide is very well adsorbed onto the root surface on perlite. That is, in 0.05 g of perlite, radish root adsorbs approximately 0.01 mg of hydrolyzed collagen peptide. Compared to similar adsorption experiments, this value shows a very good capacity both in terms of duration and the amount of amino acid retained on the surface. These results indicate that amino acids derived from dihydrolyzed collagen bind at a high level to the radish root surface. The E2 value calculated from the Dubinin–Radushkevich isotherm represents the amount of energy adsorbed per mmol of amino acid. As shown in Table 1, this value was calculated as approximately 0.032 kJ mol^−1^ (for specific equilibrium phase conditions). Accordingly, we can conclude that the adsorption process is physical. Since the Gibbs free energy calculated from the Flory–Huggins isotherm is ΔG < 0, as shown in Table 1, the adsorption is expected to occur spontaneously, and amino acid adsorption on the radish root is thought to be even stronger. The adsorption of amino acid on the root did not fit the Temkin isotherm. The regression value of the linear graph plotted for ln ce versus qe was ≤0.70, as shown in Table 1, which is quite low.

In short, among the isotherm models, the Langmuir, Freundlich, and BET models were found to show higher conformity with the experimental data. It was observed that adsorption initially occurs as a monolayer and later as a multilayer. The adsorption energy calculated from the Dubinin–Radushkevich model was determined to be approximately 0.032 kJ mol^−1^. The Gibbs free energy values obtained from the Flory–Huggins model revealed that the adsorption process is thermodynamically favorable. It was determined that the Temkin isotherm model exhibited low conformity with the experimental data.

### 3.5. Effect of Temperature on Gibbs Free Energy

Many researchers have reported that the effect of temperature influences both the adsorption isotherm and reaction kinetics during the uptake of amino acids from root and leaf tissues of plants. Among the 16 amino acids, the most frequently reported salts due to their high adsorption kinetics and solubility are L-serine, L-alanine, L-phenylalanine, L-leucine, L-cysteine, L-α-threonine, L-lysine, and L-proline. The adsorption isotherm ratios in the selected amino acid salts were measured at 298, 303, and 308 K. For comparison, the concentrations of the salts of the eight amino acids were determined in the range of 0.1–10 mM. The results are shown in Table 2. Within the temperature and concentration ranges examined, there is no inconsistency in reaction kinetics for the amino acids. In general, the same trends were observed for all selected salts.

### 3.6. Kinetic Analysis of Amino Acid Uptake by Leaf Tissue (Ex Vivo Leaf Disk Assay)

**Figure 4:**
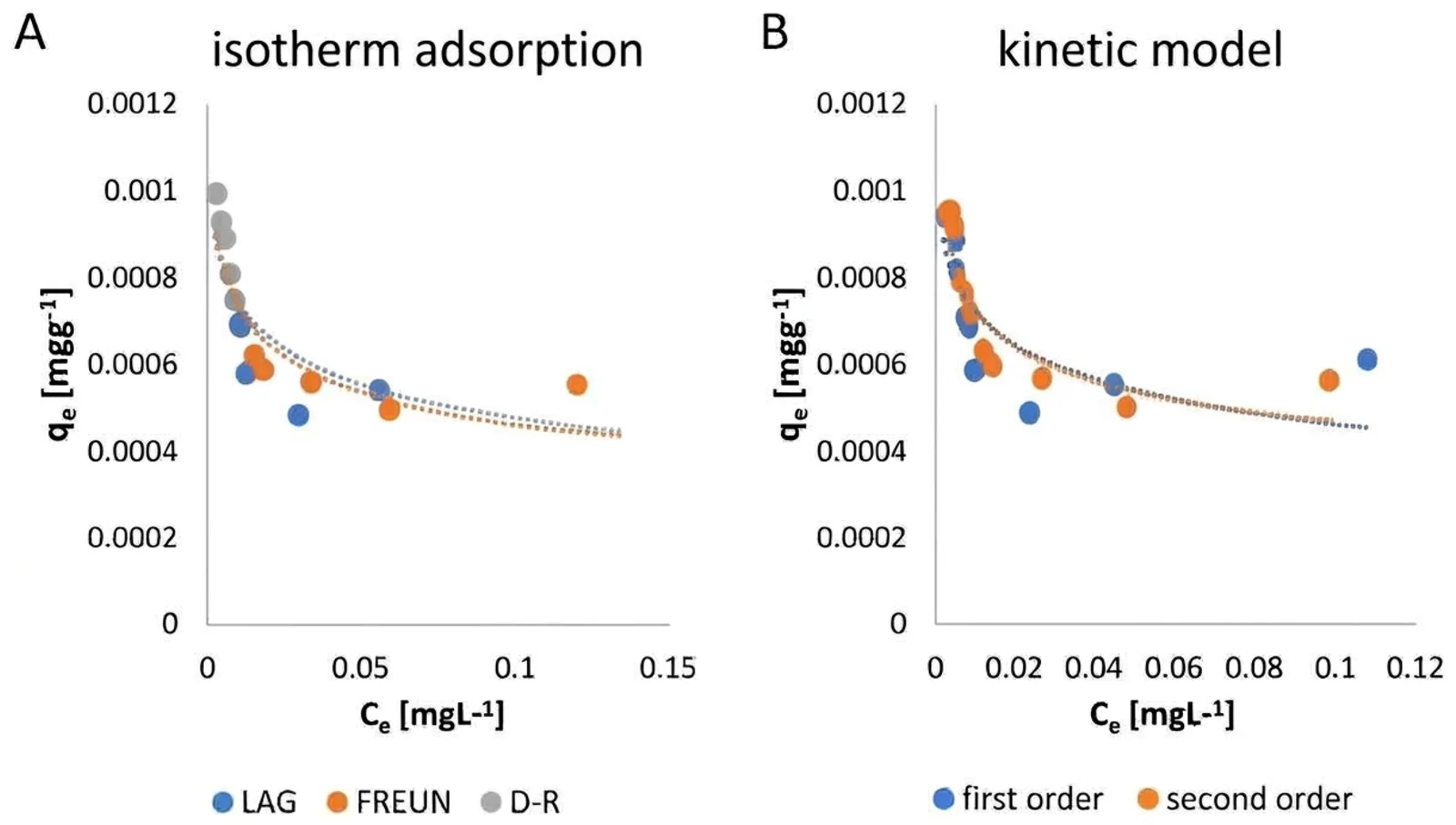
Amino acid adsorption capacity (qe) as a function of time. Freundlich, Langmuir, and D-R adsorption isotherm curves of amino acids on leaf tissue (**A**); kinetics equations of amino acids at 25 °C on leaf tissue. Pseudo-first-order, pseudo-second-order model (**B**).

In this study, three different adsorption isotherms-Langmuir, Freundlich, and Dubinin–Radushkevich (D-R)-were used to understand the adsorption behavior of dihydrolyzed collagen on the leaf tissue. The relevant equations and parameters are summarized in Table 3. Figure 4 shows the amino acid adsorption capacity (qe) as a function of time. Based on the experimental data, the adsorption isotherm was applied, and the accuracy of the correlation was evaluated by the coefficient of determination (R^2^). As shown in Figure 4 and Table 3, the Langmuir isotherm exhibited good correlation with the experimental data, and other isotherm equations also produced high R^2^ values.

The Freundlich isotherms, with the highest R^2^ value (0.9986), showed the best correlation among the isotherms introduced in this study. The constant KF of the Freundlich isotherm equation was calculated as 109.0025 L g^−1^. The 1/nF constant was obtained as 0.0596, which lies between 0 and 1, indicating a favorable adsorption process. The binding constant KS of the Dubinin–Radushkevich (D-R) isotherm model was determined to be 1.2851 L mg^−1^. Furthermore, the adsorption heat bt was calculated as 0.1836 kJ mol^−1^, demonstrating that the adsorbent–adsorbate interaction is governed by physical adsorption. The dimensionless heterogeneity factor (1/nS) of the Dubinin–Radushkevich (D-R) model was found to be 0.1762. As 1/nF approaches zero, the Dubinin–Radushkevich model approaches the Freundlich model, which indicates that overall adsorption is dominated by multilayer adsorption.

To evaluate adsorption performance, adsorption capacity was measured over time at 25 °C. After each adsorption experiment, the amino acid concentration in the leaf cells was measured and calculated as qt. Different adsorption kinetic models were applied to determine the values of the relevant parameters. The adsorption kinetic model equations studied, parameters, calculated parameter values, and R^2^ values are shown in Table 4. Among the various adsorption kinetic models examined, the pseudo-first-order model exhibited the lowest value (R^2^: 0.9739). In contrast, the pseudo-second-order model yielded the highest value (R^2^: 0.9881), indicating that the adsorption process of amino acids transported through the transport channels into leaf cells follows the pseudo-second-order adsorption model. This study shows that the uptake of amino acids by radish leaves at the tested concentrations can be adequately described using the Langmuir equation and pseudo-second-order model.

To describe the amino acid adsorption kinetics in leaf tissue, pseudo-first-order and pseudo-second-order models were applied, and the results are presented in Table 4. Both models showed a high degree of agreement with the experimental data. However, the pseudo-second-order model had a higher correlation coefficient (R^2^ = 0.9881) and explained the experimental data better than the pseudo-first-order model (R^2^ = 0.9739).

Furthermore, in the second-order model, the equilibrium capacity (qe = 135.51 mg g^−1^) was slightly higher than that of the first-order model. According to the pseudo-first-order model, the calculated equilibrium adsorption capacity (qe) was 129.14 mg g^−1^, and the rate constant (k_1_) was 1.6960 h^−1^. In the pseudo-second-order model, the qe value was calculated as 135.51 mg g^−1^, and the rate constant (k_2_) was 0.0255 g mg^−1^ h^−1^. According to the results obtained, the adsorption behavior of amino acids in leaf tissue fits the pseudo-second-order kinetic model better.

## 4. Discussion

Amino acids and small peptides such as di- and tripeptides can be taken up by plants via transporter proteins located in the plasma membrane of root cells. The uptake of larger peptides and proteins by roots can also occur indirectly through mechanisms such as apoplastic degradation and endocytosis [27,28,29]. Proteases on the root surface break down hydrolyzed proteins and convert them into amino acids, which are then taken up by membrane transporters. Some proteins can also be directly taken up via endocytosis from root hairs [30,31]. In this study, the uptake of dihydrolyzed collagen peptides by radish roots grown in perlite medium and their translocation within the plant were evaluated in the context of adsorption kinetics and isotherm models. The findings showed that roots can uptake not only inorganic nitrogen forms but also amino acids and peptides, which are low-molecular-weight organic nitrogen compounds, with high efficiency. These results support the idea that the organic nitrogen use capacity of plants is not solely dependent on symbiotic systems, but that significant contributions can be made by direct uptake through roots as well [28,31].

Transporter systems, such as amino acid permeases (AAP), proton-dependent amino acid transporters (ProT), lysine-histidine transporters (LHT), and oligopeptide transporters (OPT), have been considered to explain amino acid uptake mechanisms in plant roots. Furthermore, apoplastic and symplastic transport pathways used in the transfer of amino acids from the root epidermis to cortical tissues and long-distance transport processes via the xylem were evaluated. Previous studies on plant physiology, organic nitrogen uptake, and amino acid translocation were utilized to elucidate the transport mechanism [3,10,12,14,28].

In this study, the rapid surface adsorption observed and the kinetic behavior indicate that, rather than a single simple diffusion process at the root surface, chemical interactions and carrier-mediated mechanisms operate in conjunction in amino acid uptake. Similarly, Wyse and Komor (1984) stated that amino acid transport in plant cells is carried out by different carrier systems and that these systems exhibit substrate specificity [32]. The kinetic results obtained revealed that these carriers operate in accordance with saturation behavior and that uptake rate is concentration-dependent. The negative correlation observed in the radish plant between associated amino acid uptake capacity and charge distribution can be explained by substrate-specific transporters. Wyse and Komor (1984) identified two different amino acid carrier systems in sugarcane suspension cells: one for protonated amino acids and another for neutral amino acids [12,32]. The presence of such distinct uptake systems may account for the differing adsorption characteristics of acidic (e.g., glutamic acid and aspartic acid) and neutral amino acids. Some studies have reported that chemically diverse amino acids are transported by different carrier systems [12,33,34]. Tegeder and Masclaux-Daubresse (2018) have reported that transporter proteins are responsible for amino acid uptake by roots [3]. The same authors indicated that amino acids are transported from epidermal and cortical cells to vascular tissue via the symplastic pathway, and from there to the leaves via the xylem. This source provides the main framework for interpreting the possible translocation of collagen-derived amino acids from root to leaf in our study. Glycine exists as a polar neutral ion under appropriate pH conditions. The protonated amino group in proline and valine is fully balanced at working pH, making them zwitterions. Therefore, the acidic and neutral amino acids, which make up about 70% of the total amino acid pool [35], have low adsorption mobility in soil. In contrast, basic amino acids (e.g., arginine and lysine) will show higher affinity as they tend to bind more tightly to the cation exchange complex. At constant temperatures, the kinetic differences among amino acids are significant. Cysteine and lysine exhibit the fastest reaction kinetics. Changes in the Gibbs free energies of hydrolyzed collagen were determined at T = 298, 303, and 308 K. The activation energy values for L-alanine (40.3 ± 0.5 kJ mol^−1^), L-arginine (38.6 ± 1.0 kJ mol^−1^), and L-proline (43.0 ± 0.8 kJ mol^−1^) are in good agreement with previously reported values: 32 kJ mol^−1^ for alanine [36], and 33.3 kJ mol^−1^ for arginine. The temperature sensitivity of amino acid salts was in the order L-proline > L-arginine > L-serine > L-leucine > L-alanine > L-cysteine. According to Penny and Ritter (1983), the temperature-dependent loading capacity of amino acid salts supports the notion of surface interactions [37]. Interaction of seedling root systems with 0.1 g L^−1^, 1.0 g L^−1^, and 10 g L^−1^ hydrolyzed collagen in perlite, when calculated using the root-bioactive relationship, caused approximately a 2-fold accumulation of cysteine in root tissue after 60 min. The experiments also demonstrated that the amino acid uptake rate is strongly linked to the simultaneous presence of positive and neutral amino acids such as L-serine, L-alanine, L-phenylalanine, L-leucine, L-cysteine, L-α-threonine, L-lysine, and L-proline.

On the other hand, in our study, the uptake rate of L-glutamic acid was significantly inhibited by certain neutral amino acids. This result indicates that amino acid uptake in radish roots occurs via two distinct kinetic mechanisms. For one mechanism, positive, and for the other, neutral and negative amino acids, the uptake rates exhibited a biphasic dependence on substrate concentration. The isotherm graph is consistent with L type of Giles isotherm as seen in Figure 3. The initial tendency of the L type has shown a constant decrease with the concentration of solution and this tendency is very big. Therefore, while the adsorbate concentration is increasing, the empty spaces of the adsorbent surface decrease. Isotherm analyses indicated that the binding of amino acids to the root surface displays heterogeneous surface behavior, and it is evident that both monolayer (Langmuir-type) and multilayer (Freundlich-type) adsorption processes operate simultaneously. In the Freundlich isotherm, *n* < 1 indicates preferred adsorption [37]. High concordance with the Freundlich model suggests the system possesses energy-heterogeneous surface properties and different binding affinities [26]. Accordance with the Langmuir model supports the presence of specific binding sites and surface interactions with limited capacity [24].

The low adsorption capacity of perlite allows amino acids and peptides in solution to reach the root zone directly without being retained by the medium [19,20]. This important property offers significant advantages for research into the mechanisms of organic nitrogen compound uptake by plants. Meanwhile, amino acids taken up by roots are thought to be transported to leaves, their tissues, and cells according to enzymatic and physiological rules. The smaller the volume of an amino acid molecule taken up through carrier channels, the greater the rate of adsorption increase. Examination of molecular structure shows that the side chains of valine, isoleucine, and alanine are nonpolar. This is also true, in order of molecular chain length, for serine, proline, threonine, and glycine. That is, as the molecular volumes of amino acids decrease gradually, their adsorption rates are seen to increase. Additionally, the adsorption isotherm mechanism may be influenced by molecular polarity, since the polarity of the solvent within the carrier channels may cause electrostriction. This leads to a reduction in adsorption efficiency. During transport, phenylalanine, and other amino acids with hydrophobic side chains, can combine with only a small number of polar solvent molecules. Furthermore, molecular properties—such as side-chain charge, polarity, and molecular weight—dictate interaction efficiency; smaller, neutral, and low molecular weight amino acids (e.g., glycine, alanine, serine) exhibit higher mobility and transport rates. Crucially, all physicochemical interpretations must account for the exact ionization states of amino acid side chains at the experimental working pH of 5.8: acidic amino acids (Glu, Asp) feature fully deprotonated side-chain carboxyl groups (pI ~ 2.8–3.2), carrying a net charge of −1; basic amino acids (Lys, Arg) possess protonated side chains (pI ~ 9.7–10.8), carrying a net charge of +1; and neutral amino acids (Ala, Gly, Pro, Leu, Val, Ser, Thr, Cys) exist predominantly as zwitterions with a net charge of 0 [33]. These electrostatic and molecular distinctions directly govern their affinity toward root–surface binding sites and subsequent transport behavior. Threonine and proline are neutral polar amino acids with hydrophilic side chains. Our study indicates that, in perlite, radish roots are capable of the effective uptake of amino acids from solutions containing hydrolyzed collagen peptide at concentrations ranging from 0.1–10 g L^−1^. The low adsorption energy values obtained from the Dubinin–Radushkevich model indicate that the process is primarily characterized by physical adsorption. This suggests that amino acids temporarily bind to the root surface via electrostatic interactions and van der Waals forces, but are subsequently taken up by the cell via carrier systems. Similarly, Jones and Hodge (1999) stated that amino acids in soil solution are transported after undergoing a preliminary adsorption-based retention phase by plant roots [38]. Amino acids are kinetically taken up into leaf cells via channels by different transporters on the membrane [39]. Concentration differences among transport channels will facilitate the transfer of amino acid compounds to the leaf [40]. Depletion of amino acids from the root incubation medium followed Pseudo-First-Order (PFO) kinetics (R^2^ = 0.94–0.99), reflecting rapid apoplastic surface adsorption onto root cell walls within 60 min. In contrast, accumulation within leaf tissue conformed to the Pseudo-Second-Order (PSO) model (R^2^ = 0.9881), suggesting a carrier-mediated mechanism. The uptake efficiency of amino acids via transporter channels varies according to plant species in different ecosystems [41,42,43], environmental conditions, and amino acid type [40,44,45]. When transported from roots to leaves, amino acids with different molecular weights and positive-negative charges may exhibit multiple adsorption isotherms depending on these characteristics. It has been reported that the adsorption capacity is highest for positively charged amino acids adsorbed from leaf cells, lower for neutral amino acids, and lowest for negatively charged amino acids [38,46]. Additionally, when considering adsorption isotherms, a transporter operating at low (<5 mM) amino acid concentrations shows only slight pH dependence, whereas a transporter functioning at high (20–100 mM) amino acid concentrations is highly sensitive to external pH. Data obtained according to the adsorption kinetic model support the notion that the nature and charge properties of the amino acid transported from roots to leaves can cause an increase in substrate concentration within the leaf cells. When the hydrophobic and hydrophilic effects of amino acid side chains are considered simultaneously, the strength of the interaction with the solvent in the transport channels follows this order: glycine (or other nonpolar amino acids) <serine (or alanine) < glutamic acid < arginine < leucine < cysteine < isoleucine < proline < threonine. Therefore, as the interaction strength increases, the adsorption capacity gradually increases. At the cellular level in leaves the mesophyll tissue, which constitutes the metabolically most active and volumetrically largest portion of the leaves. Our results suggest that the parenchyma tissue, which forms the main bulk of the leaf, may play a dominant role in this uptake. Differences in amino acid uptake are closely related to molecular charge, hydrophobicity, and isomeric structures. In particular, the fact that neutral and small-molecule amino acids, such as glycine, alanine, and serine, exhibit higher transport rates indicates that membrane transporters are sensitive to substrate size and charge. This finding is consistent with the substrate selectivity of amino acid transporter proteins (AAP, LHT, and CAT families), as demonstrated by Tegeder and Masclaux-Daubresse (2018) [3].

The biphasic uptake kinetics observed in this study suggest that high- and low-affinity transporter systems operate simultaneously. Wang et al. (1993) reported that ammonium transport in plant roots exhibits a dual kinetic structure and that low-affinity transporters become predominant at high concentrations [47]. This explains the saturation trend observed in our study, particularly at high collagen concentrations. Additionally, the distribution of amino acids transported from the root to the leaves via the xylem and phloem is a key component of nitrogen remobilization within the plant. According to Paungfoo-Lonhienne et al. (2008), plants are able to directly utilise organic nitrogen derived from proteins and use this as an adaptive advantage that enables them to adapt under nitrogen-limiting conditions [31]. The results of this study support the view that collagen amino acids are not only adsorbed but also contribute to the organic nitrogen pool in leaf tissues. When the kinetic analyses of amino acid adsorption in leaf tissue were examined, the experimental data showed better conformity with the pseudo-second-order model. The high correlation coefficient (R^2^ = 0.9881) of the pseudo-second-order model suggests that the amino acid accumulation status in leaf tissues is not merely a passive process dependent on concentration gradient, but may also be related to biological uptake mechanisms involving carrier protein activation. Although isolating leaf mesophyll disks inherently disrupts the intact structural-functional continuity of the whole plant, this ex vivo approach was purposefully selected to isolate cellular transport kinetics from transpirational and xylem-phloem bulk flow variables, enabling the precise quantification of plasma-membrane-level leaf tissue cellular uptake dynamics as supported by the robust fit of this kinetic model. Therefore, this approach reflects the uptake capacity of leaf tissue rather than direct in planta delivery from roots. Amino acids are taken up by carriers located in the plasma membrane. Tegeder and Rentsch (2010) reported that the carriers involved in amino acid transport in root and leaf tissues are from the AAP (Amino Acid Permease) [14], LHT (Lysine Histidine Carrier), and ProT (Proline Carrier) families. Our findings are also consistent with the view that amino acid accumulation in leaves likely occurs via a carrier-mediated uptake mechanism. This, with the predominance of pseudo-second-order kinetics, is consistent with the results of previous studies on amino acid and organic nitrogen uptake in plant tissues. The high qe values we obtained in this study demonstrated that amino acids released from dihydrolyzed collagen are effectively retained in leaf tissue. This suggests that amino acids taken up by the roots and transported to the leaves via the xylem can form a significant organic nitrogen pool for redistribution and metabolism within the leaf. However, a limitation of this study is that root-to-shoot translocation was not traced directly; future experiments with ^15^N- or ^13^C-labelled amino acids will be needed to confirm this pathway, alongside broader evaluations of these peptide formulations [48]. Our findings reveal that the uptake of amino acids by plants is a multifactorial process jointly controlled by extracellular concentration, molecular weight, and transporter system activity. In particular, the high uptake rates of low molecular weight amino acids indicate that plants selectively utilize organic N, and this selectivity is regulated according to physiological demand [25,49].

In conclusion, dihydrolyzed collagen peptides are efficiently taken up by radish roots using physical adsorption and carrier-mediated uptake in the root surface process. Leaf disk assays showed that leaf tissue has a high capacity to take up these amino acids; their transport from roots to leaves via the vascular system remains to be confirmed by direct tracing. This physicochemical transport in our study provides important information about a robust mechanism suggesting the direct bioavailability of alternative organic nitrogen sources such as collagen by plants. By systematically differentiating between directly measured solution depletion at the root interface, modeled apoplastic adsorption, and quantified tissue accumulation, this study confirms that dihydrolyzed collagen-derived components have the potential to increase nitrogen utilization efficiency in sustainable agricultural systems.

## Figures and Tables

**Table 1:** Adsorption isotherms parameters for adsorption of amino acids on roots Image.

Freundlich *ln qe* = *ln KF* + *n ln ce*	Langmuir $\frac{\mathrm{ce}}{\mathrm{qe}}=\frac{1}{\mathrm{qmaxKL}}+\frac{\mathrm{ce}}{\mathrm{qmax}}$	BET $\frac{\mathrm{ce}}{\mathrm{qe}\left(\mathrm{cs}-\mathrm{ce}\right)}=\frac{1}{\mathrm{qm}\cdot k}+[\frac{\left(k-1\right)}{\left(\mathrm{qm}.k\right)}]\frac{\mathrm{ce}}{\mathrm{cs}}$	D-R *ln xe* = *ln xm* − *KDR ε*2 $E=\frac{1}{\sqrt{2\mathrm{KDR}}}$	F-H $\log\frac{Q}{c}=\mathrm{logKFH}+\mathrm{n log}(1-Q)$ *ΔG*0 = −2.303 *RT log KFH*
*n* 0.75 *K_F_* 1.22 *R*^2^ 0.98	*q_max_* 91.2 mg g^−1^ *K_L_* 8.0 × 10^−3^ *R*^2^ 0.92	*q_max_* 90 mg g^−1^ *R*^2^ 0.76	*q_max_* 41 mg g^−1^ *K_DR_* 8.0 × 10^−5^ *E* 0.091 kJ mol^−1^ *R*^2^ 0.69	*K_FH_* 4.2 × 10^−5^ ΔG^0^ 30 kJ mol^−1^ *R*^2^ 0.75

**Table 2:** Evaluation of collagen adsorption in root tissue at different temperatures by absorbance measurements.

Temperature (Root)	Abs. (Unexposed)	Abs. (Collagen-Exposed)
25 °C	1.09	0.571
30 °C	1.09	0.503
35 °C	1.09	0.485

**Table 3:** Adsorption isotherm parameters for amino acid uptake on leaf tissue.

Dubinin–Radushkevich (D-R)	Freundlich	Langmuir	Isotherm Adsorption Models
qm = 139.3951 mg g^−1^	KF = 109.0025 L g^−1^	qm = 200 mg g^−1^	
KL = 11.9509 L mg^−1^	nF = 15.9490	1/nS = 0.1762	
RL = 0.0003–0.0021	1/nF = 0.0596	KS = 1.2851 L mg^−1^	
R^2^ = 0.9534	R^2^ = 0.9986	R^2^ = 0.9974	

**Table 4:** Kinetic model parameters for amino acid adsorption on leaf tissue.

Pseudo-First- Order	Pseudo-Second-Order	Adsorption Kinetic Models
qe = 129.1423 mg g^−1^	qe = 135.5145 mg g^−1^	
k_1_ = 1.6960 h^−1^	k_2_ = 0.0255 g mg^−1^ h^−1^	
R^2^ = 0.9739	R^2^ = 0.9881	

## Data Availability

The data presented in this study are available within the article.

## References

[1] Zhang J., Liu Y., Liu Y., Zhang S., Zhang S., Zeng H., Ding W., Zhang D., Xu G. (2026). Plant nitrogen nutrition: Enhancing plant resilience to abiotic stresses. J. Genet. Genom..

[2] Bloom A.J. (2015). The increasing importance of distinguishing among plant nitrogen sources. Curr. Opin. Plant Biol..

[3] Tegeder M., Masclaux-Daubresse C. (2018). Source and sink mechanisms of nitrogen transport and use. New Phytol..

[4] Wang Z. (2026). Amino Acids as Multifunctional Molecules in Plants: From Fundamental Metabolism to Precision Agriculture. Plants.

[5] Lipson D., Näsholm T. (2001). The unexpected versatility of plants: Organic nitrogen use and availability in terrestrial ecosystems. Oecologia.

[6] Lee Y.H., Foster J., Chen J., Voll L.M., Weber A.P.M., Tegeder M. (2007). AAP1 transports uncharged amino acids into roots of Arabidopsis. Plant J..

[7] Ma A., Xun W., Zhang S., Liang S., Wei W., Huang H., Shen Q., Xu G., Zhang R. (2026). Amino-acid-transporter-mediated assembly of rhizosphere microbiota enhances soil organic nitrogen acquisition in rice. Nat. Plants.

[8] Colla G., Nardi S., Cardarelli M., Ertani A., Lucini L., Canaguier R., Rouphael Y. (2015). Protein hydrolysates as biostimulants in horticulture. Sci. Hortic..

[9] Forsum O., Svennerstam H., Ganeteg U., Näsholm T. (2008). Capacities and constraints of amino acid utilization in Arabidopsis. New Phytol..

[10] Yao X., Nie J., Bai R., Sui X. (2020). Amino acid transporters in plants: Identification and function. Plants.

[11] Dong K., Ye Z., Hu F., Shan C., Wen D., Cao J. (2026). Corrigendum to “Improvement of plant quality by amino acid transporters: A comprehensive review”. Plant Physiol. Biochem..

[12] Rentsch D., Schmidt S., Tegeder M. (2007). Transporters for uptake and allocation of organic nitrogen compounds in plants. FEBS Lett..

[13] Liu L., Yu X., Yan Y., He C., Wang J., Sun M., Li Y. (2024). Amino acid transporters in amino acid absorption, transport and distribution in crops. Horticulturae.

[14] Tegeder M., Rentsch D. (2010). Uptake and partitioning of amino acids and peptides. Mol. Plant.

[15] Taiz L., Zeiger E., Møller I.M., Murphy A. (2015). Plant Physiology and Development.

[16] Rouphael Y., Colla G. (2020). Biostimulants in agriculture. Front. Plant Sci..

[17] Al Hajj W., Hussein M.S., Hassan F., Krayem M., Khati S.E. (2024). Hydrolyzed collagen: Exploring its applications in the food and beverage industries and assessing its impact on human health—A comprehensive review. Heliyon.

[18] León-López A., Morales-Peñaloza A., Martínez-Juárez V.M., Vargas-Torres A., Zeugolis D.I., Aguirre-Álvarez G. (2019). Hydrolyzed collagen: Sources and applications. Molecules.

[19] Aziz A., Bellil A., El Amrani El Hassani I., Fekhaoui M., Achab M., Dahrouch A., Benzaouak A. (2021). Geopolymers based on natural perlite and kaolinitic clay from Morocco: Synthesis, characterization, properties, and applications. Ceram. Int..

[20] Gruda N. (2019). Increasing Sustainability of Growing Media Constituents and Stand-Alone Substrates in Soilless Culture Systems. Agronomy.

[21] Kang J.N., Kim J.S., Lee S.M., Won S.Y., Seo M.S., Kwon S.J. (2021). Analysis of phenotypic characteristics and sucrose metabolism in the roots of *Raphanus sativus* L. Front. Plant Sci..

[22] Ochar K., Ki D.W., Moon S., Bissah M.N., Kim S.H. (2026). Quantitative analysis of root system architecture and fresh weight biomass traits highlight phenotypic variation in radish (*Raphanus sativus* L.) germplasm. Sci. Rep..

[23] Freundlich H. (1906). Über die Adsorption in Lösungen. Z. Phys. Chem..

[24] Langmuir I. (1918). The adsorption of gases on plane surfaces of glass, mica and platinum. J. Am. Chem. Soc..

[25] Misra D.N. (1969). Adsorption on heterogeneous surfaces: A Dubinin–Radushkevich equation. Surf. Sci..

[26] Carriere P.P.E., Reed B.E., Cline S.R. (1995). Retention and release of lead by a silty loam and a fine sandy loam. II. Kinetics. Sep. Sci. Technol..

[27] Fan L., Li R., Pan J., Ding Z., Lin J. (2015). Endocytosis and its regulation in plants. Trends Plant Sci..

[28] Näsholm T., Kielland K., Ganeteg U. (2009). Uptake of organic nitrogen by plants. New Phytol..

[29] Thanthrige N., Lawrence N., Craik D.J. (2026). Biotechnological approaches for producing therapeutic peptides in plants. npj Sci. Plants.

[30] Dhanasekaran P., Chittibabu S., Mouzeyar S., Boulaflous-Stevens A., Delattre C., Roche J. (2025). From waste to wonder: The potential of protein hydrolysates as plant biostimulants in agriculture. Bioresour. Technol. Rep..

[31] Paungfoo-Lonhienne C., Lonhienne T.G., Rentsch D., Robinson N., Christie M., Webb R.I., Gamage H.K., Carroll B.J., Schenk P.M., Schmidt S. (2008). Plants can use protein as a nitrogen source without assistance from other organisms. Proc. Natl. Acad. Sci. USA.

[32] Wyse R.E., Komor E. (1984). Mechanisms of amino acid uptake by sugar cane suspension cells. Plant Physiol..

[33] Fischer W.N., Loo D.D., Koch W., Ludewig U., Boorer K.J., Tegeder M., Rentsch D., Wright E.M., Frommer W.B. (2002). Low and high affinity amino acid H+-cotransporters for cellular import of neutral and charged amino acids. Plant J..

[34] Yang G., Wei Q., Huang H., Xia J. (2020). Amino Acid Transporters in Plant Cells: A Brief Review. Plants.

[35] Kielland K. (1990). Factors Controlling Nitrogen Release and Turnover in Arctic Tundra. Ph.D. Thesis.

[36] Kim M., Song H.J., Lee M.G., Jo H.Y., Park J.W. (2012). Kinetics and steric hindrance effects of carbon dioxide absorption into aqueous potassium alaninate solutions. Ind. Eng. Chem. Res..

[37] Penny D., Ritter T.J. (1983). Kinetic study of the reaction between carbon dioxide and primary amines. J. Chem. Soc. Faraday Trans. 1.

[38] Jones D.L., Hodge A. (1999). Biodegradation kinetics and sorption reactions of three differently charged amino acids in soil and their effects on plant organic nitrogen availability. Soil Biol. Biochem..

[39] Anraku Y., Payne J.W. (1980). Transport and utilization of amino acids by bacteria. Microorganisms and Nitrogen Sources.

[40] Sauheitl L., Glaser B., Weigelt A. (2009). Uptake of intact amino acids by plants depends on soil amino acid concentrations. Environ. Exp. Bot..

[41] McKane R.B., Johnson L.C., Shaver G.R., Nadelhoffer K.J., Rastetter E.B., Fry B., Giblin A.E., Kielland K., Kwiatkowski B.L., Laundre J.A. (2002). Resource-based niches provide a basis for plant species diversity and dominance in arctic tundra. Nature.

[42] Warren C.R. (2009). Does nitrogen concentration affect relative uptake rates of nitrate, ammonium, and glycine?. J. Plant Nutr. Soil Sci..

[43] Warren C.R., Adams P.R. (2007). Uptake of nitrate, ammonium and glycine by plants of Tasmanian wet eucalypt forests. Tree Physiol..

[44] Thornton B., Robinson D. (2005). Uptake and assimilation of nitrogen from solutions containing multiple N sources. Plant Cell Environ..

[45] Rothstein D.E. (2010). Effects of amino-acid chemistry and soil properties on the behavior of free amino acids in acidic forest soils. Soil Biol. Biochem..

[46] Vinolas L.C., Healey J.R., Jones D.L. (2001). Kinetics of soil microbial uptake of free amino acids. Biol. Fertil. Soils.

[47] Wang M.Y., Siddiqi M.Y., Ruth T.J., Glass A.D.M. (1993). Ammonium uptake by rice roots. II. Kinetics of 13NH4+ influx across the plasmalemma. Plant Physiol..

[48] Karcioglu B.L., Yavas C., Hekim N. (2025). Kiperin double-hydrolyzed collagen as potential anti-tumor agent: Effects on HCT116 colon carcinoma cells and oxidative stress modulation. Curr. Issues Mol. Biol..

[49] Kielland K. (1994). Amino acid absorption by arctic plants: Implications for plant nutrition and nitrogen cycling. Ecology.

